# *eNOS* polymorphisms and clinical outcome in advanced HCC patients receiving sorafenib: final results of the ePHAS study

**DOI:** 10.18632/oncotarget.8569

**Published:** 2016-04-04

**Authors:** Andrea Casadei Gardini, Giorgia Marisi, Luca Faloppi, Emanuela Scarpi, Francesco Giuseppe Foschi, Massimo Iavarone, Gianfranco Lauletta, Jody Corbelli, Martina Valgiusti, Floriana Facchetti, Cristina della Corte, Luca Maria Neri, Stefano Tamberi, Stefano Cascinu, Mario Scartozzi, Dino Amadori, Oriana Nanni, Elena Tenti, Paola Ulivi, Giovanni Luca Frassineti

**Affiliations:** ^1^ Department of Medical Oncology, Istituto Scientifico Romagnolo per lo Studio e la Cura dei Tumori (IRST) IRCCS, Meldola, Italy; ^2^ Biosciences Laboratory, Istituto Scientifico Romagnolo per lo Studio e la Cura dei Tumori (IRST) IRCCS, Meldola, Italy; ^3^ Department of Medical Oncology, Azienda Ospedaliero Universitaria Ospedali Riuniti, Università Politecnica delle Marche, Ancona, Italy; ^4^ Unit of Biostatistics and Clinical Trials, Istituto Scientifico Romagnolo per lo Studio e la Cura dei Tumori (IRST) IRCCS, Meldola, Italy; ^5^ DPT Internal Medicine, Faenza Hospital, AUSL Romagna, Faenza, Italy; ^6^ A.M.&A. Migliavacca Center for Liver Disease, 1st Division of Gastroenterology, Fondazione IRCCS Ca' Granda Maggiore Hospital, University of Milan, Milan, Italy; ^7^ Department of Biomedical Sciences and Human Oncology, Internal Medicine “G. Baccelli”, University of Bari “A. Moro”, Bari, Italy; ^8^ Department of Medical Oncology, Faenza Hospital, AUSL Romagna, Faenza, Italy; ^9^ Department of Morphology, Surgery and Experimental Medicine, University of Ferrara, Ferrara, Italy; ^10^ Department of Medical Oncology, University Hospital Cagliari, Cagliari, Italy

**Keywords:** hepatocellular carcinoma, endothelial nitric oxide synthase, single nucleotide polymorphisms, biomarkers, angiogenesis

## Abstract

Sorafenib may reduce endothelial nitric oxide synthase (eNOS) activity by inhibiting vascular endothelial growth factor receptors (VEGF-R), leading to a decrease in nitric oxide production. In the Italian multicenter ePHAS (*eNOS* polymorphisms in HCC and sorafenib) study, we analyzed the role of *eNOS* polymorphisms in relation to clinical outcome in patients with hepatocellular carcinoma (HCC) receiving sorafenib. Our retrospective study included a training cohort of 41 HCC patients and a validation cohort of 87 HCC patients, all undergoing sorafenib treatment. Three *eNOS* polymorphisms (*eNOS* -786T>C, *eNOS* VNTR 27bp 4a/b and *eNOS*+894G>T) were analyzed by direct sequencing or Real Time PCR in relation to progression-free survival (PFS) and overall survival (OS) (log-rank test). In univariate analysis, training cohort patients homozygous for *eNOS* haplotype (HT1:T-4b at *eNOS*-786/*eNOS* VNTR) had a lower median PFS (2.6 *vs.* 5.8 months, *P* < 0.0001) and OS (3.2 *vs.*14.6 months, *P* = 0.024) than those with other haplotypes. In the validation set, patients homozygous for HT1 had a lower median PFS (2.0 *vs.* 6.7 months, *P* < 0.0001) and OS (6.4 *vs.*18.0 months, *P* < 0.0001) than those with other haplotypes. Multivariate analysis confirmed this haplotype as the only independent prognostic factor. Our results suggest that haplotype HT1 in the *eNOS* gene may be capable of identifying a subset of HCC patients who are resistant to sorafenib.

## INTRODUCTION

Hepatocellular carcinoma (HCC) represents the most common primary liver cancer and is increasing in incidence [1]. The introduction of sorafenib has changed the clinical landscape of the disease, showing modest efficacy and reasonable tolerability [2–4]. Markers of sorafenib efficacy or resistance have yet to be identified [5–7].

The inhibition of VEGFR-2 by sorafenib is known to repress phosphoinositide 3-kinase (PI3K) and its downstream serine protein kinase (Akt), decreasing the activity of endothelium-derived nitric oxide synthase (eNOS) and reducing the production of the potent vasodilator nitric oxide (NO) [8–10]. NO, constitutively expressed by vascular endothelial cells, controls a variety of physiologic functions including neovascularization, angiogenesis [8, 9, 11] and pathological conditions [12, 13]. In particular, it appears to play a proangiogenic role in tumor angiogenesis [14].

Numerous studies have reported that specific *eNOS* single nucleotide polymorphisms (SNPs) affect gene transcription, resulting in a variation in eNOS protein levels and activity and consequently influencing NO [15, 16].

Among known polymorphisms, *eNOS*-786 T>C in the promoter region, a 27bp variable number of tandem repeats in intron 4 (*eNOS* VNTR 4a/b) and *eNOS+*894 G>T in exon 7 have received the greatest attention [15, 17, 18]. Numerous studies have investigated the extent to which *eNOS* polymorphisms influence the risk of developing cancer [19–21] and cardiovascular diseases [22–26], with conflicting results. However, it is still unclear how these polymorphisms affect gene expression and enzyme activity in cells and how they influence response to anti-angiogenic drugs [27]. The aim of the ePHAS study (*eNOS* polymorphisms in HCC and sorafenib) was to evaluate the prognostic value of *eNOS* polymorphisms in two independent cohorts of advanced HCC patients undergoing treatment with sorafenib.

## RESULTS

### Patient characteristics

The main clinical pathological characteristics of patients are shown in Table 1. In the training cohort the median follow-up was 50 months (range 1-82). Median progression-free survival (PFS) was 3.9 months (95% CI 2.7-5.7) and median overall survival (OS) was 11.3 months (95% CI 6.7-14.9). The dose of sorafenib was reduced in 9 (21.9%) patients. The median follow-up in the validation cohort was 47 months (range 1-52). Median PFS was 4.6 (95% CI 2.6-5.7), while median OS was 12.4 months (95% CI 8.2-17.2). Seventeen (19.5%) patients required a reduction in the dose of sorafenib. Clinical pathological characteristics were similar between the two cohorts, with the exception of median age and etiology.

**Table 1:** Patient characteristics

Clinical and pathologic indexes	Training cohort (n=41)	Validation cohort (n=87)	
	No. of Patients (%)	No. of Patients (%)	p
**Median age, years (range)**	72 (28-87)	67 (24-86)	0.016
**Gender**			
Male	32 (78.1)	60 (69.0)	
Female	9 (21.9)	27 (31.0)	0.392
**Smoking habits**			
No	18 (50.0)	12 (42.9)	
Yes	18 (50.0)	16 (57.1)	0.752
**Etiology**			
Metabolic syndrome	8 (19.5)	5 (5.7)	
Alcohol	6 (14.6)	6 (6.9)	
Viral	27 (65.8)	71 (81.7)	
Cryptogenic	0	5 (5.7)	0.017
**BCLC stage**
B	8 (20.0)	20 (25.9)	
C	32 (80.0)	57 (74.1)	0.624
**MELD score**			
≤10	30 (81.1)	37 (62.7)	
>10	7 (18.9)	22 (37.3)	0.093
**Serum α-FP level**			
≤400 KUI/L	20 (52.6)	52 (69.3)	
>400 KUI/L	18 (47.4)	23 (30.7)	0.124
**Diabetes**			
No	24 (60.0)	68 (78.2)	
Yes	16 (40.0)	19 (21.8)	0.056
**Sorafenib dose reduction**			
No	32 (78.1)	70 (80.5)	
Yes	9 (21.9)	17 (19.5)	0.753
**Portal vein thrombosis**			
No	21 (70.0%)	39 (67.2%)	
Yes	9 (30.0%)	19 (32.8%)	0.982
**Liver cirrhosis**			
No	1 (2.4%)	2 (2.2%)	
Yes	40 (97.6%)	85 (97.8%)	0,954
**Disease extension**			
Liver only	23 (69.6%)	42 (72.4%)	
Metastatic disease	7 (30.4%)	16 (27.6%)	0.861

BCLC: Barcelona-Clinic Liver Cancer; MELD: Model For End-Stage Liver Disease

### Clinical variables

PFS and OS data in relation to baseline patient characteristics and toxicity in both cohorts are shown in Table 2. In particular, we found that validation cohort patients with a MELD score ≤10 showed better PFS (5.7 *vs.* 1.6 months, *P* < 0.0001) and OS (13.6 *vs.* 4.4 months, *P* = 0.004) than those with a MELD score > 10. These data were not significant in the training cohort. With regard to hypertension, training cohort patients with increased systolic blood pressure (> 140 mmHg) and/or increased diastolic blood pressure (> 90 mmHg) after 15 days' treatment with sorafenib showed better PFS (6.1 *vs.* 2.8 months, *P* = 0.005) and OS (14.6 *vs.*7.5 months, *P* = 0.027) than those with no hypertension. No data on hypertension were available for the validation cohort.

**Table 2:** PFS and OS in relation to clinical characteristics and toxicity in the two independent cohorts

	Training set							Validation set						
	No. of Patients	No. of Events	Median PFS (95% CI)	*P*	No. of Events	Median OS (95% CI)	*P*	No. of Patients	No. of Events	Median PFS (95% CI)	*P*	No. of Events	Median OS (95% CI)	*P*
**Gender**														
Male	32	31	3.7 (2.6-4.7)		28	8.7 (3.9-14.9)		60	50	3.9 (2.4-6.2)		43	11.3 (8.2-17.2)	
Female	9	7	8.2 (2.2-11.2)	0.166	7	14.6 (6.6-23.0)	0.266	27	23	5.0 (2.1-6.3)	0.516	19	14.1 (5.6-27.0)	0.842
**Smoking habits**														
No	18	17	4.3 (2.6-8.5)		16	10.8 (6.8-14.6)		12	9	13.8 (2.1-27.1)		6	27.8 (14.1-35.0)	
Yes	18	17	4.2 (2.6-6.2)	0.997	17	9.8 (2.9-15.8)	0.578	16	11	2.4 (1.1-15.2)	0.112	9	5.2 (2.5-39.0)	0.125
**Etiology**														
Metabolic syndrome	8	8	3.0 (0.9-6.0)		6	6.8 (1.0-nr)		5	4	2.4 (2.1-8.7)		3	7.5 (3.7-9.9)	
Alcohol	6	6	6.7 (2.6-34.2)		6	15.3 (2.7-34.2)		6	5	13.5 (2.0-31.4)		5	14.4 (3.0-50.8)	
Viral	27	24	4.7 (2.6-5.8)		23	11.2 (6.9-15.1)		71	59	3.9 (2.5-5.7)		50	12.4 (8.2-18.0)	
Cryptogenic	0	0	-	0.195	0	-	0.458	5	5	3.4 (1.7-27.0)	0.599	4	10.5 (2.5-nr)	0.729
**BCLC stage**														
B	8	7	4.3 (0.5-21.6)		6	10.0 (0.5-23.0)		11	6	9.0 (4.8-27.1)		2	18.0 (14.4-nr)	
C	32	30	3.8 (2.7-5.7)	0.780	28	12.0 (6.7-14.9)	0.747	38	30	2.5 (2.0-8.7)	0.084	24	19.5 (3.7-28.8)	0.100
**MELD score**														
≤10	30	29	3.9 (2.6-6.0)		26	12.0 (3.9-14.9)		37	32	5.7 (3.9-12.8)		29	13.6 (9.7-27.8)	
>10	7	6	3.8 (1.2-34.2)	0.706	6	8.5 (2.9-34.2)	0.863	22	22	1.6 (1.1-2.4)	<0.0001	20	4.4 (2.5-10.9)	0.004
**Serum α-FP level**														
≤400 KUI/L	20	18	3.8 (2.6-6.0)		16	10.1 (3.9-23.6)		52	44	5.0 (3.4-6.3)		38	12.4 (7.2-19.5)	
>400 KUI/L	18	17	4.6 (2.6-10.8)	0.327	16	12.0 (3.9-14.9)	0.809	23	20	2.4 (1.3-4.8)	0.044	18	7.5 (2.5-13.9)	0.043
Hypertension^*^														
No	21	20	2.8 (2.2-3.9)		19	7.5 (3.2-14.9)		-	0	-		0	-	
Yes	18	17	6.1 (3.7-10.8)	0.005	15	14.6 (11.2-23.0)	0.027	-	0	-	-	0	-	
**Skin toxicity**														
No	23	21	3.8 (2.3-5.2)		19	6.9 (3.2-14.9)		53	43	2.8 (2.4-5.6)		34	13.6 (6.1-17.2)	
Yes	18	17	6.2 (2.6-10.8)	0.065	16	13.9 (7.5-16.7)	0.124	34	30	5.3 (3.7-12.8)	0.035	28	11.3 (7.2-28.6)	0.349
**Diarrhea**														
No	35	32	3.8 (2.6-5.8)		29	10.4 (6.7-14.9)		65	52	3.7 (2.5-6.2)		45	10.9 (6.4-18.0)	
Yes	6	6	5.0 (2.7-21.6)	0.447	6	13.9 (2.7-23.6)	0.876	22	21	5.0 (2.1-9.9)	0.365	17	13.9 (10.5-28.8)	0.301
**Asthenia**														
No	28	27	3.9 (2.6-5.3)		26	10.8 (5.2-14.9)		86	72	4.7 (2.6-5.7)		61	12.4 (8.2-17.2)	
Yes	13	11	4.7 (1.9-8.7)	0.681	9	13.7 (3.9-16.7)	0.997	1	1	2.1 (−)	0.281	1	3.0 (−)	0.045
**Mucositis**														
No	40	37	3.8 (2.6-5.7)		34	11.2 (6.7-14.9)		81	68	4.6 (2.6-5.7)		57	11.8 (8.2-17.2)	
Yes	1	1	15.3 (−)	0.397	1	15.8 (−)	0.762	6	5	4.8 (1.1-nr)	0.439	5	9.8 (2.2-nr)	0.917

PFS, progression-free survival; OS, overall survival; BCLC, Barcelona-Clinic Liver Cancer; MELD, Model For End-Stage Liver Disease

* diastolic pressure >90 mmHg or systolic pressure >140 mmHg recorded 15 days after the start of sorafenib treatment

### *eNOS* genotypes and haplotype analysis

*eNOS-*786 and *eNOS+*894 genotypes were successfully determined in all of the samples. *eNOS* VNTR genotype was not evaluable in 3 samples (2 in the training set and one in the validation set) because of their poor quality.

Genotype frequencies of *eNOS*-786, *eNOS* VNTR and *eNOS*+894 are shown in Supplementary Table 1 and all genotype frequencies followed the Hardy-Weinberg equilibrium. We observed a linkage disequilibrium between *eNOS*-786 and *eNOS* VNTR in both the training and validation cohorts (correlation coefficient, r^2^ = 0.227; D'= 0.85 and r^2^ =0.172; D'= 0.746, respectively) and identified a total of 4 haplotypes (HT). The most frequent haplotype in either cohort was HT1 (T-4b at *eNOS*-786/*eNOS* VNTR) (58.1% in the training cohort and 62% in the validation cohort), followed by HT2 (C-4b) (24.2% and 23.4%, respectively), HT3 (C-4a) (16% and 12.2%, respectively) and HT4 (T-4a), this last occurring at a frequency of < 5% (1.6% and 2.4%, respectively).

### *eNOS* genotypes and clinical outcome in the training cohort

**Figure 1:**
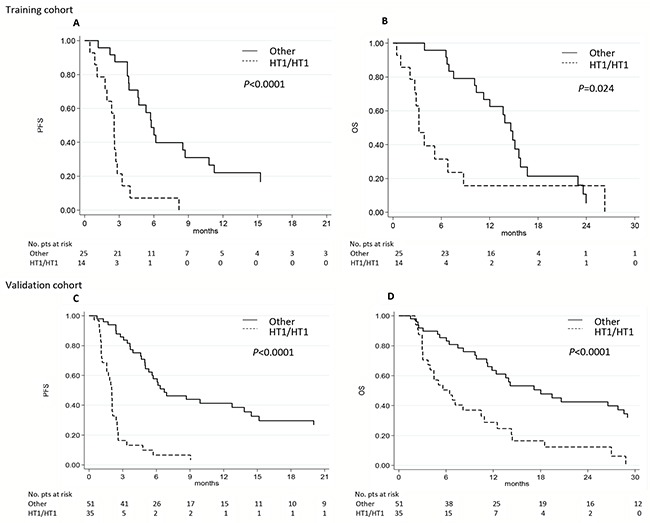
eNOS haplotypes and clinical outcome in the two independent cohorts **A-C.** Progression-free survival (PFS) and **B-D.** overall survival (OS) in relation to *eNOS* haplotypes (HT) in the training and validation cohorts. Other, haplotypes other than the one indicated.

In univariate analysis we found that all 3 SNPs were associated with PFS but not with OS (Table 3 and Supplementary Figure 1). *eNOS*-786 TT, *eNOS* VNTR 4bb and *eNOS*+894 GG genotypes were significantly associated with a lower median PFS (2.6, 2.8 and 2.8 months, respectively) than other genotypes (5.8, 8.5, and 5.5 months, respectively) (*P* = 0.0001, *P* = 0.046 and *P* = 0.049, respectively). Interestingly, patients homozygous for HT1 had a lower median PFS than those with other haplotypes (2.6 *vs.* 5.8 months, *P* < 0.0001) (Table 3 and Figure 1A).

**Table 3:** Univariate analysis of PFS according to *eNOS* polymorphisms in the training and validation cohorts

	Training cohort					Validation cohort				
	No. of Patients	No. of Events	Median PFS (95% CI)	HR (95% CI)	*P*	No. of Patients	No. of Events	Median PFS (95% CI)	HR (95% CI)	*P*
**Overall**		38	3.9 (2.7-5.7)	-	-		73	4.6 (2.6-5.7)	-	-
***eNOS*-786**										
TT	15	15	2.6 (1.1-2.8)	4.43 (2.08-9.42)	0.0001	37	35	2.0 (1.6-2.1)	5.81 (3.43-9.82)	<0.0001
CC+TC	26	23	5.8 (3.8-8.7)	1.00		50	38	6.9 (5.6-14.5)	1.00	
***eNOS* VNTR**										
4bb	26	25	2.8 (2.3-4.6)	2.08 (1.01-4.29)	0.046	62	52	2.5 (2.1-4.8)	1.97 (1.18-3.31)	0.010
4ab+4aa	13	11	8.5 (3.8-15.3)	1.00		24	21	6.1 (4.7-23.9)	1.00	
***eNOS*+894**										
GG	18	16	2.8 (2.3-3.8)	2.00 (1.00-3.99)	0.049	42	40	2.5 (2.0-3.7)	2.17 (1.35-3.49)	0.001
GT+TT	23	22	5.5 (3.7-8.5)	1.00		45	33	6.9 (4.8-12.8)	1.00	
*eNOS* Haplotypes ^*^ (786/VNTR)										
HT1/HT1	14	14	2.6 (1.1-2.8)	5.43 (2.46-11.98)	<0.0001	35	33	2.0 (1.6-2.1)	5.16 (3.06-8.68)	<0.0001
Other	25	22	5.8 (3.8-8.7)	1.00		51	40	6.7 (5.0-13.8)	1.00	

PFS, progression-free survival; OS, overall survival; HR, hazard ratio.

* Haplotypes (HT) 1 shows the allele T at *eNOS*-786 and allele 4b at *eNOS* VNTR. “Other” indicates haplotypes other than the one indicated.

With regard to OS, only patients homozygous for haplotype HT1 had a lower median OS than those with other haplotypes (3.2 *vs.*14.6 months, respectively; *P* = 0.024) (Table 4 and Figure 1B).

**Table 4:** Univariate analysis of OS according to *eNOS* polymorphisms in the training and validation cohorts

	Training cohort				Validation cohort			
	No. of Events	Median OS (95% CI)	HR (95% CI)	*P*	No. of Events	Median OS (95% CI)	HR (95% CI)	*P*
**Overall**	35	11.3 (6.7-14.9)	-	-	62	12.4 (8.2-17.2)	-	-
***eNOS*-786**								
TT	13	3.9 (2.1-8.7)	1.85 (0.91-3.77)	0.088	29	6.4 (3.7-10.5)	3.41 (1.96-5.95)	<0.0001
CC+TC	22	14.6 (10.4-15.8)	1.00		33	19.5 (12.4-29.0)	1.00	
***eNOS* VNTR**								
4bb	22	6.9 (3.2-14.9)	1.21 (0.57-2.59)	0.621	44	10.5 (6.4-14.1)	1.76 (1.00-3.09)	0.048
4ab+4aa	11	14.6 (10.1-15.8)	1.00		18	19.5 (8.2-31.5)	1.00	
***eNOS*+894**								
GG	13	7.5 (3.2-16.7)	1.38 (0.67-2.84)	0.387	35	7.5 (4.9-17.2)	1.79 (1.07-2.99)	0.027
GT+TT	22	12.8 (6.8-15.6)	1.00		27	14.1 (11.2-27.0)	1.00	
*eNOS* Haplotypes ^*^ (786/VNTR)								
HT1/HT1	12	3.2 (2.1-6.8)	2.35 (1.12-4.91)	0.024	27	6.4 (3.7-10.5)	3.01 (1.73-5.23)	<0.0001
Other	21	14.6 (10.4-15.8)	1.00		35	18.0 (11.3-28.6)	1.00	

PFS, progression-free survival; OS, overall survival; HR, hazard ratio.

* Haplotypes (HT) 1 shows the allele T at *eNOS*-786 and allele 4b at *eNOS* VNTR. “Other” indicates haplotypes other than the one indicated.

Following adjustment for clinical covariates (age, gender, etiology, BCLC stage, serum α-FP level and MELD score), multivariate analysis confirmed *eNOS*-786 and the specific haplotype of *eNOS* gene as the only independent prognostic factors predicting PFS (HR 10.24, 95%CI 2.88-36.45, *P* = 0.0003; HR 9.76, 95%CI 3.19-29.85, *P* < 0.0001, respectively) and OS (HR 4.98, 95%CI 1.48-16.69, *P* = 0.009; HR 2.64, 95%CI 1.10-6.34, *P* = 0.03, respectively) (Table 5). These data remained statistically significant after Bonferroni correction.

**Table 5:** Multivariate analysis in the training and validation cohorts

	Training cohort				Validation cohort			
	PFS		OS		PFS		OS	
	HR (95% CI)	*P*	HR (95% CI)	*P*	HR (95% CI)	*P*	HR (95% CI)	*P*
***eNOS*-786**								
CT+CC	1.00		1.00		1.00		1.00	
TT	10.24 (2.88-36.45)	0.0003	4.98 (1.48-16.69)	0.009	5.87 (1.28-26.99)	0.023	0.56 (0.04-8.29)	0.677
***eNOS* VNTR**								
4ab+4aa	1.00		1.00		1.00		1.00	
4bb	0.83 (0.31-2.21)	0.703	0.46 (0.16-1.29)	0.141	3.31 (0.67-16.31)	0.141	7.04 (0.70-70.73)	0.097
***eNOS*+894**								
GT+TT	1.00		1.00		1.00		1.00	
GG	1.16 (0.48-2.79)	0.741	0.59 (0.21-1.64)	0.309	1.48 (0.44-5.04)	0.528	11.95 (1.15-24.12)	0.038
*eNOS* Haplotypes^*^								
Other	1.00		1.00		1.00		1.00	
HT1/HT1	9.76 (3.19-29.85)	<0.0001	2.64 (1.10-6.34)	0.030	11.17 (3.71-33.63)	<0.0001	7.03 (1.86-26.55)	0.004

PFS, progression-free survival; OS, overall survival; HR, hazard ratio.

* HT1 shows the allele T at *eNOS*-786 and allele 4b at *eNOS* VNTR. “Other” indicates haplotypes other than the one indicated.

### *eNOS* genotypes and clinical outcome in the validation cohort

In univariate analysis, we confirmed that all 3 SNPs were associated with PFS (Table 3 and Supplementary Figure 2A–2C). *eNOS*-786 TT, *eNOS* VNTR 4bb and *eNOS*+894 GG were significantly associated with a lower median PFS (2.0, 2.5 and 2.5 months, respectively) than the other genotypes (6.9, 6.1 and 6.9 months, respectively) (*P* < 0.0001, *P* = 0.01 and *P* = 0.001, respectively). We also confirmed that patients homozygous for HT1 had a lower median PFS than those with other haplotypes (2.0 *vs.* 6.7 months, *P* < 0.0001) (Table 3 and Figure 1C). These results remained statistically significant after Bonferroni correction.

With regard to OS, *eNOS*-786 TT, *eNOS* VNTR 4bb and *eNOS*+894 GG genotypes were significantly associated with a lower median OS (6.4, 10.5 and 7.5 months, respectively) than the other genotypes (19.5, 19.5 and 14.1 months, respectively) (*P* = 0.0001, *P* = 0.048, *P* = 0.027, respectively) (Table 4 and Supplementary Figure 2D–2F). We also found that patients homozygous for haplotype HT1 had a lower median OS than those with other haplotypes (6.4 *vs.*18.0 months, respectively, *P* < 0.0001) (Table 4 and Figure 1D). This result remained statistically significant after Bonferroni correction.

Following adjustment for clinical covariates (age, gender, etiology, BCLC stage, serum α-FP level and MELD score), multivariate analysis confirmed the *eNOS* haplotype as the only independent prognostic factor predicting PFS (HR 11.17, 95%CI 3.71-33.63, *P* < 0.0001) and OS (HR 7.03, 95%CI 1.86-26.55, *P* = 0.004). These data remained statistically significant after Bonferroni correction (Table 5). Furthermore, no significant associations were observed between *eNOS* polymorphisms and hypertension, skin toxicity, asthenia, mucositis or diarrhea in the validation cohort (data not shown).

### *eNOS* genotypes and objective response rate (ORR) in the training and validation cohort

*eNOS* polymorphisms were also investigated in relation to ORR (Supplementary Table 2). In the training cohort, patients carrying a TT genotype for *eNOS*-786 showed a higher percentage of progression at the first CT re-evaluation than those carrying other genotypes (76.9% *vs*. 34.8%, respectively) (*P* = 0.013). These data were confirmed in the validation cohort (55.6% *vs*. 3.7%, respectively) (*P* < 0.0001).

In the training cohort, patients carrying homozygous HT1 showed a higher percentage of progression at the first CT re-evaluation than those carrying other haplotypes (76.9% *vs*. 31.8%, respectively) (*P* = 0.009). These data were confirmed in the validation group (57.7% *vs*. 3.7%, respectively) (*P* < 0.0001). We also observed that patients homozygous for haplotype HT1 in either cohort showed a lower percentage of complete and partial response (0% and 3.8, respectively) than those carrying other haplotypes (18.2% and 18.5%, respectively) at the first CT re-evaluation.

## DISCUSSION

Our study of 2 independent cohorts (training and validation) revealed that advanced HCC patients homozygous for a specific *eNOS* haplotype showed the worst PFS and OS. Few biomarkers predicting drug response are available in clinical practice for many cancer types [28, 29] and as far as we know this is the first study to demonstrate the role of *eNOS* polymorphisms in relation to clinical outcome in advanced HCC patients receiving sorafenib.

We found that patients homozygous for *eNOS* haplotype in most cases showed disease progression at the first CT re-evaluation. Moreover, patients with other genotypes associated with a better PFS and OS showed higher response rates.

Several clinical trials have been performed on the combined use of transarterial chemoembolization (TACE) and sorafenib [4, 30–34]. However, these studies did not succeed in their primary aim because patients were not selected on the basis of molecular markers.

The results obtained from our analysis of *eNOS* polymorphisms suggest that they could identify potential candidates for treatment with combination therapies including TACE-sorafenib and could help to evaluate the efficacy of sorafenib in patients without good liver function (Child-Pugh B).

In the literature, only a few studies have identified possible markers of response to sorafenib in HCC patients. Post-hoc analysis of the SHARP study demonstrated that low baseline plasma concentrations of VEGF-A and angiopoietin-2 were associated with better OS [6, 35], but this has not been confirmed by other authors. Polymorphism analysis seems to have more advantages than protein or gene expression analysis. Gene expression analysis is performed on biological material collected at a specific time in the natural history of the disease. It is also subject to the influence of a number of laboratory biases. Conversely, polymorphism analysis can be performed at any time during the course of the disease, is not substantially influenced by laboratory biases and is less expensive. In this regard, only one study on polymorphisms and response to sorafenib showed that *VEGF-A* and *VEGF-C* polymorphisms were independent factors influencing PFS and OS [36].

Previous studies suggested that DNA variants at the *eNOS* gene can quantitatively control *eNOS* expression [25, 37]. The point variation at nucleotide-786bp has been associated with a significant reduction in *eNOS* gene promoter activity, resulting in lower levels of eNOS mRNA, eNOS protein and enzyme activity [24, 37]. With regard to the variable number tandem repeat, the rare allele 4-repeat homozygote shows the highest eNOS mRNA levels, which are, however, associated with lower eNOS protein levels and enzyme activities [24, 37]. It has also been suggested that this polymorphism modulates eNOS expression though the formation of small RNAs (sirRNAs). Endothelial cells containing 5 repeats show higher quantities of sirRNA and lower levels of eNOS mRNA when compared with cells containing 4 repeats [38, 39]. In addition, *eNOS*+894G>T variation in exon 7 of the *eNOS* gene, leading to an amino acid change from Glu to Asp (Glu298Asp), is associated with reduced eNOS protein levels, enzyme activities and basal NO production [40, 41]. Moreover, Wang et al. demonstrated that the functional 27-bp repeat at intron 4 coordinates with the *eNOS*-786 variant and may directly affect transcription efficiency [25].

In our study, TT homozygotes for the *eNOS*-786 variant, allele 5-repeat homozygotes for *eNOS* VNTR and GG homozygotes for +894 variant resulted in lower PFS and OS. In agreement with previous studies, these kinds of variants seem to be associated with higher eNOS protein levels and enzyme activities, and consequently with increased basal NO production. We therefore hypothesized an association between high levels of eNOS protein/activity and sorafenib resistance.

With regard to toxicity, we found that patients with hypertension during sorafenib treatment showed better PFS and OS, as previously observed [42, 43]. An increased in blood pressure seems to be closely related to eNOS. The activation of VEGFR-2 also stimulates the production of NO and inhibits endothelin-1 (ET-1), a potent vasoconstrictor [44, 45]. In patients treated with sorafenib, inhibition of VEGFR-2 may reduce NO, resulting in vasoconstriction and hypertension.

The main strength of our multicenter study is that the analyses were performed on two independent cohorts of patients. Moreover, patients in the validation cohort were treated by different specialists (oncologist, gastroenterologist and hepatologist). The study also has a number of limitations, *e.g.* its retrospective nature (cases were, however, consecutively selected, thus reducing potential bias). Thus, we were only able to collect data on hypertension for the training cohort. In a previous work we found that the early onset of hypertension was associated with improved clinical outcome in HCC patients treated with sorafenib [46]. Given the possible correlation between *eNOS* polymorphisms and hypertension [27], it would have been interesting to evaluate this in our validation cohort. As our study was carried out on white individuals only, our findings cannot be automatically extrapolated to patients of other ethnicities. Another limitation of our study is the absence of a control arm not receiving sorafenib. Thus, a clear distinction cannot be made between the prognostic and predictive role of *eNOS* polymorphisms in relation to survival.

In conclusion, the presence of a specific haplotype of *eNOS*-786 and *eNOS* VNTR polymorphisms may identify a subset of HCC patients who are more resistant to sorafenib. These data now require confirmation in a prospective study.

## MATERIALS AND METHODS

### Patients and treatment

This retrospective multicenter Italian study was conducted on a training cohort of 41 HCC patients consecutively treated at Istituto Scientifico Romagnolo per lo Studio e la Cura dei Tumori from 2012 to 2014. A retrospective validation cohort of 87 HCC patients was consecutively recruited by four other participating centers (Faenza Hospital and the Universities of Ancona, Milan and Bari) from 2012 to 2015.

Patients receiving sorafenib with advanced- or intermediate-stage HCC (either histologically proven or diagnosed according to the AASLD [American Association for the Study of Liver Diseases 2005] guidelines) refractory or no longer amenable to locoregional therapies, were eligible for our analysis. Eligibility criteria were the same as those of Llovet's pivotal study on sorafenib in HCC: [3] Eastern Cooperative Oncology Group (ECOG) performance status score ≤2; Child-Pugh liver function class A; adequate hematologic function (platelet count, ≥60×10^9^/L; hemoglobin ≥8.5 g/dL; and prothrombin time international normalized ratio ≤2.3 or prothrombin time ≤6 seconds above control, adequate hepatic function (albumin ≥2.8 g/dL; total bilirubin ≤3 mg/dL [51.3 μmol/L]; alanine aminotransferase and aspartate aminotransferase ≤5 times the upper limit of the normal range); and adequate renal function (serum creatinine ≤1.5 times the upper limit of the normal range).

All patients received sorafenib according to the standard schedule (400 mg bid continuously), dose reductions applied when clinically indicated. Follow-up consisted of a CT/MRI scan every 8 weeks or as clinically indicated. Tumor response was evaluated by modified Response Evaluation Criteria in Solid Tumors (mRECIST) [47]. Treatment with sorafenib was continued until disease progression, unacceptable toxicity or death.

Hypertension was defined as an increase in systolic blood pressure (> 140 mmHg) and/or in diastolic blood pressure (> 90 mmHg) after 15 days' treatment with sorafenib. The Model For End-Stage Liver Disease (MELD) score cutoff was 10 [48]. The study was approved by the Local Ethics Committees of each center and informed consent was obtained from each patient for their biological material to be used for research purposes.

### DNA isolation and genotyping

On the basis of our previous results confirming that *eNOS* polymorphism analysis is feasible regardless of the starting material used [49], we performed *eNOS* genotyping using DNA extracted from whole blood or formalin-fixed paraffin-embedded (FFPE) HCC tissue. For peripheral blood samples collected in EDTA tubes, genomic DNA was extracted from 200 μl of whole blood by QIAamp DNA Minikit (Qiagen SPA, Milan, Italy) in accordance with the manufacturer's instructions. For tissue samples, paraffin wax was removed with xylene and samples were washed twice with 100% ethanol. DNA was isolated from the deparaffinized tissue using the Recover-All™ Total Nucleic Acid Isolation Kit for FFPE Tissues (Applied Biosystems, Foster City, CA) in accordance with the manufacturer's instructions. DNA quantity and quality were assessed by Nanodrop 1000 (Celbio, Milan, Italy).

SNPs in the *eNOS* gene are well documented polymorphisms and were selected after a review of the medical literature. *eNOS*-786 T>C (rs2070744) is located in 5′ promoter region, *eNOS* VNTR 27bp 4a/b in intron 4 and *eNOS*+894G>T (rs1799983) in exon 7. *eNOS* VNTR 27bp 4a/b in intron 4 has 2 common alleles: “4a” with 4 repeats and “4b” with 5 repeats.

Genotyping analyses of *eNOS*-786 and *eNOS*+894 were performed by TaqMan technology using SNP genotyping assays. Polymerase chain reaction (PCR) was performed and genotypes were analyzed on the 7500 Real-Time PCR System (Applied Biosystems) using a 7500 Software version 2.3. PCRs were performed starting from 20 ng of genomic DNA. Conversely, *eNOS* VNTR was determined by standard PCR and direct sequencing analysis on an ABI 3130 Genetic Analyser (Applied Biosystems). PCR conditions and primer sequences for *eNOS* VNTR were reported in our previous study [49]. All samples were analyzed at the same institution (Biosciences Laboratory, IRST IRCCS, Meldola, Italy).

### Statistical analysis

Hardy-Weinberg equilibrium, linkage disequilibrium and haplotype analyses were performed using the Haploview program version 4.2 [50]. This software provides Lewontin's disequilibrium coefficient (D') as the measure of the nonrandom association of alleles at different loci. The D' coefficient is equal to 1 only if 2 SNPs have not been separated by recombination (or recurrent mutation) during the history of the sample (complete degree of linkage disequilibrium [LD]).

The primary objective of this study was to evaluate the prognostic value of *eNOS* polymorphisms in relation to clinical outcome (PFS and OS) in a cohort of advanced HCC patients undergoing sorafenib treatment (training cohort). The second objective was to verify whether *eNOS* polymorphisms are related to objective response. The prognostic value of *eNOS* polymorphisms in patients with advanced HCC was then confirmed in an independent cohort (validation cohort).

PFS was defined as the time from the first administration of sorafenib until the first report of objective disease progression or death due to any cause, whichever occurred first, or until the date of the last follow-up. OS was defined as the time from the first administration of sorafenib until death due to any cause, or until the date of the last follow-up. Event-time distributions were estimated using the Kaplan-Meier method and survival curves were compared using the log-rank test.

Cox proportional hazard ratios were identified separately for each polymorphism. The significance threshold for an overall type I error rate of 0.05 was set at *P* < 0.0062 based on a conservative Bonferroni correction for multiple comparison. We subsequently analyzed significant polymorphisms identified in this step using Cox regression analysis adjusting for baseline covariates (age, gender, etiology, Barcelona-Clinic Liver Cancer [BCLC] stage, serum α-FP level and MELD score).

The association between polymorphisms and objective response (OR, defined as complete/partial response *vs.* stable disease *vs.* progressive disease) was examined using the Chi-Square test with a significance level of *P* = 0.05. All statistical analyses were performed using SAS Statistical Software (version 9.3, SAS Institute Inc., Cary, NC). All *P* values were two-sided.

## SUPPLEMENTARY FIGURES AND TABLES



## Abbreviations

HCC: hepatocellular carcinoma
eNOS: endothelial nitric oxide synthase
NO: nitric oxide
SNP: single nucleotide polymorphism
VNTR: variable number tandem repeat
PFS: progression free survival
OS: overall survival
ePHAS: eNOS polymorphisms in HCC and sorafenib
