# Supplementary material for: eNOS polymorphisms and clinical outcome in advanced HCC patients receiving sorafenib: final results of the ePHAS study
Source: Oncotarget. 2016 Apr 4;7(19):27988–99. doi: 10.18632/oncotarget.8569 (PMC5053704; doi:10.18632/oncotarget.8569)
Supplement: Supplementary file 1 [file oncotarget-07-27988-s001.pdf]

SUPPLEMENTARY FIGURES AND TABLES

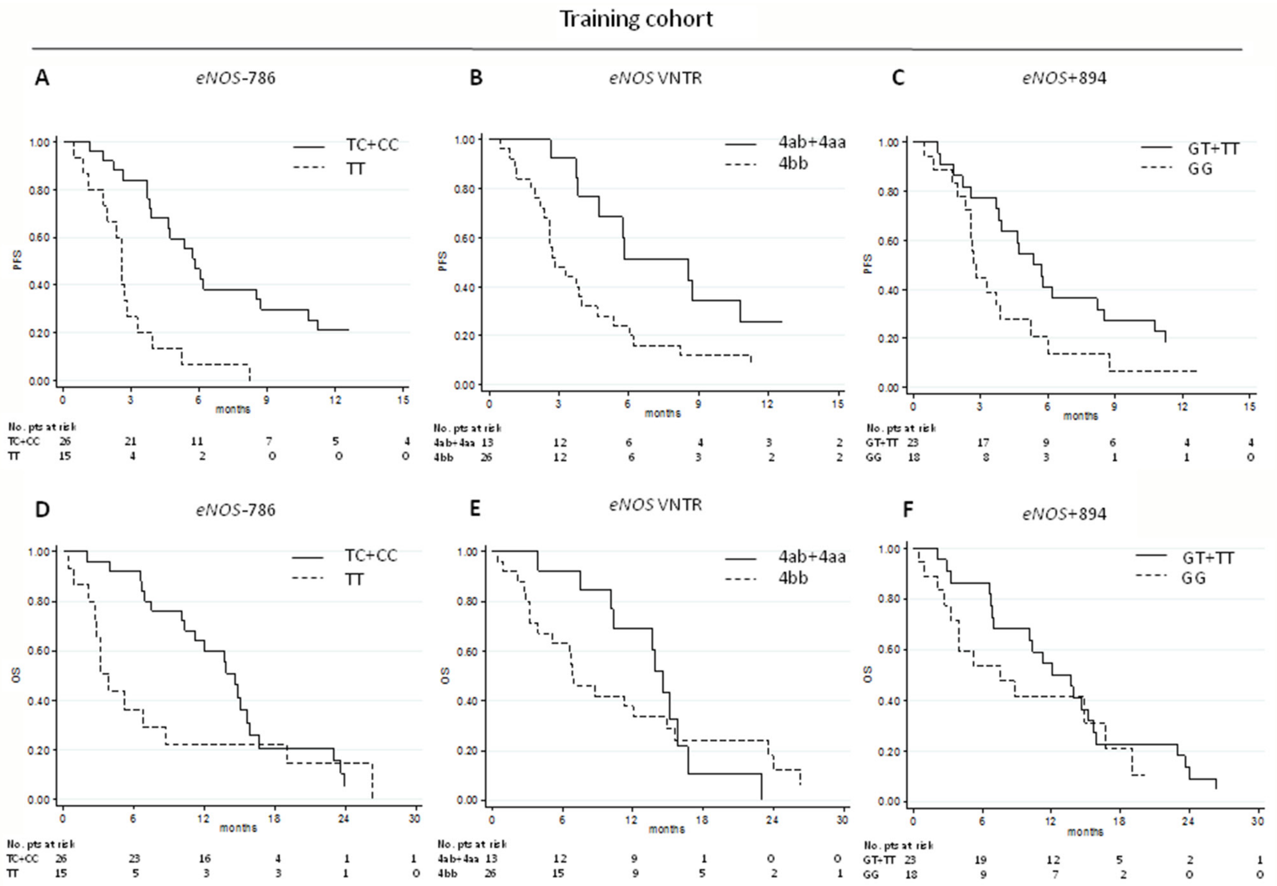

Supplementary Figure 1: eNOS polymorphisms and clinical outcome in the training cohort. Progression-free survival (PFS) and overall survival (OS) in relation to *eNOS*-786 **A.** and **D.** respectively), *eNOS* VNTR **B.** and **E.** respectively) and *eNOS*+894 (**C.** and **F.** respectively).

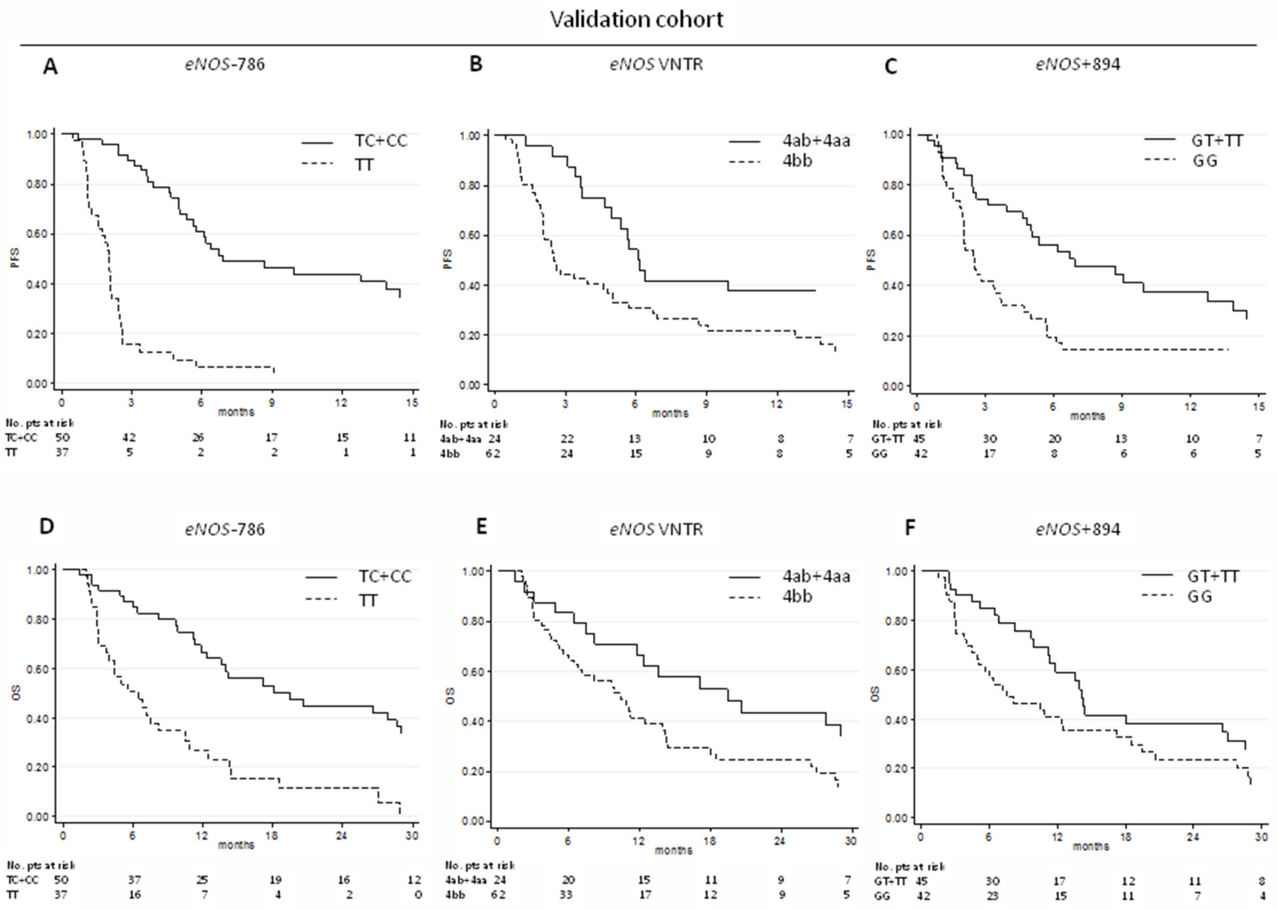

Supplementary Figure 2: eNOS polymorphisms and clinical outcome in the validation cohort. Progression-free survival (PFS) and overall survival (OS) in relation to *eNOS*-786 **A.** and **D.** respectively), *eNOS* VNTR **B.** and **E.** respectively) and *eNOS*+894 **C.** and **F.** respectively).

Supplementary Table S1: *eNOS* genotype frequencies in the training and validation cohort

eNOS SNPs	Training cohort (n = 41)		Validation cohort (n = 87)	
	No. of patients (%)	Hardy-Weinberg equilibrium	No. of patients (%)	Hardy-Weinberg equilibrium
***eNOS* -786 T>C**				
CC	7 (17.1)	$P = 0.53$	12 (13.8)	$P = 0.78$
TC	19 (46.3)		38 (43.7)	
TT	15 (36.6)		37 (42.5)	
***eNOS* VNTR**				
4bb	26 (66.7)	$P = 0.74$	62 (72.1)	$P = 0.89$
4ab	12 (30.8)		23 (26.7)	
4aa	1 (2.5)		1 (1.2)	
n.e.	2		1	
***eNOS* +894 G>T**				
GG	18 (43.9)	$P = 0.71$	42 (48.3)	$P = 0.89$
GT	22 (53.7)		36 (41.4)	
TT	1 (2.4)		9 (10.3)	

SNP, single-nucleotide polymorphism; VNTR, variable number tandem repeat; n.e., not evaluable

Supplementary Table S2: Association between *eNOS* genotypes and ORR

SNPs	No. of patients	CR/PR No. (%)	SD No. (%)	PD No. (%)	*P*
**Testing set**					
***eNOS*-786**					
TT	13	0	3 (23.1)	10 (76.9)	
TC/CC	23	4 (17.4)	11 (47.8)	8 (34.8)	0.013
***eNOS* VNTR**					
4bb	23	2 (8.7)	7 (30.4)	14 (60.9)	
4ab/4aa	12	2 (16.7)	7 (58.3)	3 (25.0)	0.074
***eNOS*+894**					
GG	16	1 (6.2)	6 (37.5)	9 (56.3)	
GT/TT	20	3 (15.0)	8 (40.0)	9 (45.0)	0.386
***eNOS* Haplotype**					
HT1/HT1	13	0	3 (23.1)	10 (76.9)	
Other	22	4 (18.2)	11 (50.0)	7 (31.8)	0.009
**Validation set**					
***eNOS*-786**					
TT	27	1 (3.7)	11 (40.7)	15 (55.6)	
TC/CC	27	5 (18.5)	21 (77.8)	1 (3.7)	< 0.0001
***eNOS* VNTR**					
4bb	40	5 (12.5)	20 (50.0)	15 (37.5)	
4ab/4aa	13	1 (7.7)	11 (84.6)	1 (7.7)	0.208
***eNOS*+894**					
GG	28	2 (7.1)	15 (53.6)	11 (39.3)	
GT/TT	26	4 (15.4)	17 (65.4)	5 (19.2)	0.092
***eNOS* Haplotype**					
HT1/HT1	26	1 (3.8)	10 (38.5)	15 (57.7)	
Other	27	5 (18.5)	21 (77.8)	1 (3.7)	< 0.0001
